# Life on holidays: differences in activity composition between school and holiday periods in Australian children

**DOI:** 10.1186/s12889-019-6765-6

**Published:** 2019-06-03

**Authors:** Tim Olds, Carol Maher, Dorothea Dumuid

**Affiliations:** 1 Alliance for Research in Exercise, Nutrition and Activity (ARENA), University of South Australia, Adelaide, Australia; 2 Murdoch Children’s Research Institute, Melbourne, Australia

**Keywords:** Use of time, Compositional data analysis, Screen time, Sleep, Physical activity

## Abstract

**Background:**

Recently, a small number of studies have suggested that gains in fitness and reductions in body fat achieved during the school term are reversed or stagnate during the holiday period. This may be associated with changed activity patterns. The aim of this study was to compare 24-h activity compositions between school and holiday periods in Australian children.

**Methods:**

The participants in this study were 366 children (53% female, 13.4 ± 2.3 years) who were a subgroup of the 2007 Australian National Children’s Nutrition and Physical Activity Survey. Each child recalled use of time on at least one school day, one weekend day and one holiday using the Multimedia Activity Recall for Children and Adults. Composite “in-term” and “holiday” use-of-time profiles were generated by weighting school days by 5, and weekends by 2 where data were available. Difference between holiday and in-term time use was assessed using a compositional multivariate linear model for repeated measures. Subsequent models tested for interaction between time of measurement and socio-economic status or body mass index.

**Results:**

Time use was significantly different between holidays and in-term days (F = 103, *p* < 0.0001). On holidays, children accumulated 140 min less School-related time, compensated by sleeping 40 min longer, 58 min more Screen Time, and 35 min more Domestic/Social time. Children spent 10 min less in vigorous physical activity, and although sitting time was 33 min/day less during holidays, estimated total daily energy expenditure (TDEE) was 5.4% lower. Differences between holiday and in-term activity compositions did not vary by parental education (F = 1.2, *p* = 0.25), postcode-level socio-economic status (F = 0.9, *p* = 0.56) or weight status (F = 1.7, *p* = 0.07).

**Conclusions:**

In this subsample of a nationally representative survey of Australian children, holidays were characterised by longer sleep and higher TV and videogame time, lower vigorous activity and lower TDEE. Uncompensated by dietary adjustments, these differences would result in an accumulation of about 650 g of fat over a six-week holiday period. Holiday activity patterns may be a promising focus for obesity prevention efforts.

**Electronic supplementary material:**

The online version of this article (10.1186/s12889-019-6765-6) contains supplementary material, which is available to authorized users.

## Background

Children’s activity patterns have wide-ranging impacts on almost all domains of their lives, including their physical and psychosocial health, school and motor performance, and wellbeing [1]. Despite these benefits, Australian children’s physical activity (PA) levels are declining [2], and few children meet screen time guidelines. The prevalence of childhood overweight and obesity has quadrupled since the 1960s [3], and aerobic fitness has declined in recent decades [4]. Childhood fatness and low fitness are associated with poorer adult health and higher mortality [5, 6]. A variety of interventions (predominantly school-based) have targeted children’s lifestyle patterns with limited success [7].

Children are exposed to a range of environments — school, community, home —which may facilitate or limit increases in fatness or declines in fitness. Over the year, children spend about 15% of their time at school, but 25% of the entire year is spent on holidays. The holiday break constitutes a major hiatus in learning, and a body of literature has documented relative declines in cognitive skills over the holiday period (the so-called *summer learning loss*; [8]. Losses are greater among children from disadvantaged families [9], such that the gap grows ever larger over successive years.

More recently, a small number of studies, mainly from North America, have addressed whether changes in fitness and fatness across the summer holiday period show the same pattern. The major finding has been that gains in fitness and reductions in body fat achieved during the school term are reversed or stagnate during the summer holidays [10], with some groups (e.g. ethnic minorities [11] and obese children [12]) being particularly affected. A recent study in a nationally representative sample of 18,170 US children tracked from kindergarten to Grade 2 found that *all* the increases in fatness (BMI) occurred over the summer break [13]. Similarly, in a study of 3588 Texas elementary school children followed for six years, BMI percentile fell by 1.5 points during school term, but increased 5.2 points during the summer holidays [14].

There have been similar findings for fitness in European children. In a cohort of 178 Greek children, the monthly rate of improvement in the shuttle run test was about half as great over the summer holidays as during the in-term period [15]. A recent report from the *ukactive* Research Institute [16] found that the average shuttle run performance of 400 UK children fell from 740 m at the end of term to 606 m after the summer holidays. Several intervention studies have found similar summer holiday losses of in-term improvements in body fat, fitness and fasting insulin [17].

These changes might be explained by differences in how children use their time over the holiday period compared to term time. Very few studies have tracked time use across the holiday period, and findings are mixed. A doubly-labeled water study [18] found 2.4% lower total daily energy expenditure (TDEE) during holidays (1.66 METs) than in school term (1.70 METs) in overweight American 6–13 year olds, though the difference was not significant. Other studies have found *higher* PA in summer [19]. Staiano et al. [20] also found that school-aged children were generally more active on holidays than during school term, though this was coupled with 30 min/day more TV time and 12 min/day more computer time on holidays, so the overall effect on TDEE is hard to quantify.

Preserving in-school levels of healthy activity during the holiday period is particularly important because of a somewhat counter-intuitive asymmetry in the effects of reducing and increasing healthy behaviours. Recent compositional data analyses have demonstrated that decreasing moderate-to-vigorous physical activity (MVPA) has greater detrimental health consequences than the benefits achieved by increasing MVPA by the same amount [21], largely due to the nonlinear and approximately logarithmic dose-response relationship between PA and health outcomes [22]. In 7000 10–11 year old children from 12 countries, 30 min/day more MVPA was associated with approximately a 0.35 unit decrease in BMI z-score, whereas 30 min/day less PA was associated with an increase of over 0.6 units — almost twice as great [21]. If these cross-sectional associations reflect longitudinal dependencies, it is more important to defend than to increase current levels of physical activity. The reduction in MVPA (and accompanying increase in screen time and sitting) associated with the holiday period may be particularly deleterious, and should perhaps be the focus of our efforts.

## Methods

### Aim

The aim of the present study was to describe the activity patterns of children from a nationally representative Australian sample during in-term (i.e. school day and school-time weekend day) and holiday periods. Our hypotheses were (a) that activity compositions would differ between the two periods; and (b) these differences would be greater for children from low socio-economic backgrounds and for overweight or obese children.

### Participants

The sample for this study was drawn from the Australian National Children’s Nutrition and Physical Activity Survey (NCNPAS), a nationally representative survey of 4458 children aged 5–16 years conducted in 2007 [23], of whom 2200 aged 9–16 completed time-use recalls. Children were recruited by random-digit dialing, with an overall response rate of 40%. This study used NCNPAS data from a subgroup of 366 children who recalled at least one holiday, one school day, and one weekend day during the school term.

### Measurements

Height and weight were measured by trained interviewers using Invicta Height Measure stadiometers and Tanita HD332 electronic scales. Body mass index was calculated from height and weight, and converted to a z-score using the UK 1990 reference dataset. Children were classified as normal weight/underweight (*n* = 259), overweight (*n* = 76), or obese (*n* = 28), and BMI z-scores were calculated using the International Obesity Task Force criteria [24]. To quantify socio-economic status (SES), two parent-reported measures were used. The Socio-economic Indicators for Areas Index of Relative Disadvantage (SEIFA IRSD) is a standardised postcode-level measure of SES based on a basket of measures such as education, income and employment. The IRSD has a national mean of 1000 and a standard deviation of 100, with higher values indicating less disadvantage. Parents also reported the highest level of education of either parent, which was collapsed into one of three categories: university (*n* = 162), some post-secondary (*n* = 140), or high school (*n* = 61). IRSD and parental education values were not available for three participants. The three missing IRSD values were imputed by multivariate imputation using chained equations, via the R package *mice* [25]. The predictive mean matching method was used, based on complete data for parental education level and child zBMI.

Use of time was assessed using the Multimedia Activity Recall for Children and Adults (MARCA; [26]). Children recalled everything they did from wake-up to bedtime over four days across two separate computer-assisted telephone interviews, using a segmented-day format with a resolution of 5 min or more. The MARCA has been validated against accelerometry [26]), pedometry [27], and doubly-labeled water [28], with validity coefficients of r_s_ = 0.45–0.70. Test-retest reliability is very high (ICC = 0.84–0.92; [26]). The 259 individual activities in the MARCA are hierarchically aggregated into 13 “macrodomains” and eight “superdomains” (Table 1).

**Table 1:** The MARCA activity hierarchy

Superdomain	Macrodomain	Examples
Domestic/Social	Social	Sitting talking
	Chores/Work	Cleaning room
Passive Transport		Riding in a car or bus
Physical Activity	Sport	Soccer
	Play	Playground games
	Active Transport	Riding a bicycle
Quiet Time		Listening to music
School-related	Classroom	Writing sitting
	Study/Homework/Music	Playing the piano
	Reading	Reading *War and Peace*
Screen Time	TV	Watching *Game of Thrones*
	Computer	Surfing the Internet
	Videogames	X-Box
Self-care	Eating	Eating sitting
	Grooming	Showering, towelling off
Sleep		Sleeping including naps

Four days of data were collected for each child in the course of 2007. Each child was interviewed twice by telephone, each time recalling the previous day and the day before. For the 366 children in this sub-sample, at least one of these days was a school day, one a weekend day during the school term, and one a holiday. If there were two days of one type (e.g. two school days), one was randomly chosen for analysis. On average, holidays were recalled 0.5 days earlier than in-term days (median absolute difference = 17 days). Not all holiday periods refer to summer holidays, but could be any holiday throughout the year. From these data, two “composite days” were constructed. From the days occurring in school term (i.e. the school day and the in-term weekend day), an average day was generated, using a 5:2 weighting for weekdays:weekend days. The In-term day therefore represented a weighted amalgam of school and non-school days during the term period.

Each activity in the MARCA is linked to a compendium of energy expenditures [29] so that overall and activity-specific energy costs can be estimated. Like the Ainsworth compendium, the Ridley compendium is based on existing empirical data and comparisons with cognate activities. For certain physical activities, such as sports, respondents are asked to indicate whether their effort was hard, medium or light, and are given cues such as breathing and heart rate. These were used to estimate total daily energy expenditure (TDEE, in MET.min) using the factorial method (i.e. the time spent in each activity was multiplied by the rate of energy expenditure derived from the Ridley compendium, and divided by 1440 min/day). Time spent in sedentary behaviours (operationalised as activity requiring ≤1.5 METs while awake and sitting or lying down), light physical activity (LPA; 1.6–2.9 METs), moderate physical activity (MPA; 3.0–5.9 METs), and vigorous physical activity (VPA; ≥6 METs) was also calculated.

### Statistical analysis

Statistical analysis was performed in R [30] using the following packages; *compositions* [31], *zCompositions* [32] and *car* [33]. Daily time use in the MARCA superdomains and macrodomains was described for in-term and holiday periods using arithmetic means and standard deviations. Activity compositions were compared at the superdomain level using compositional data analysis (CoDA). According to CoDA procedures, compositions were represented as coordinates in real Euclidean space via an isometric log ratio (*ilr*) transformation. The eight-part compositions were expressed as a set of seven *ilrs*.

Repeated-measures MANOVA was used, following the procedures outlined in O’Brien and Kaiser [34]. Fourteen *ilrs* (two sets of seven *ilrs* for each participant) were considered as response variables. Time of measurement (holiday, in-term time) and the *ilr* number were considered as main effects, with test for interaction to check if the set of *ilrs* (or time-use composition) changed across time of measurement. Subsequent models tested for additional interactions with socio-economic or BMI categories.

## Results

Participant characteristics are shown in Table 2. The subgroup was not significantly different from the larger NCNPAS sample in terms of age (mean for both 13.4 years), BMI z-score (both + 0.54), % overweight or obesity (subgroup mean 29% vs 26% for NCNPAS), SEIFA (1003 vs 1002), % female (53 vs 51), geographical distribution (56% vs 54% living in a major city), or educational characteristics of parents (44% vs 40% university-educated).

**Table 2:** Participant characteristics

	Boys	Girls	All
n (%)	172 (47)	194 (53)	366
Age (years)	13.4 (2.3)	13.4 (2.3)	13.4 (2.3)
BMI (kg/m^2^)	20.3 (4.0)	21.2 (4.1)	20.8 (4.1)
BMI z-score	0.51 (1.24)	0.57 (1.15)	0.54 (1.19)
% overweight/obese	27	30	29
SEIFA IRSD	1005 (66)	1001 (70)	1003 (68)

Values shown are means (SDs) unless indicated

*BMI* Body mass index, *IRSD* Index of Relative Social Disadvantage, *SEIFA* Socio-economic Indicators for Areas

### Differences in time use

**Fig. 1:**
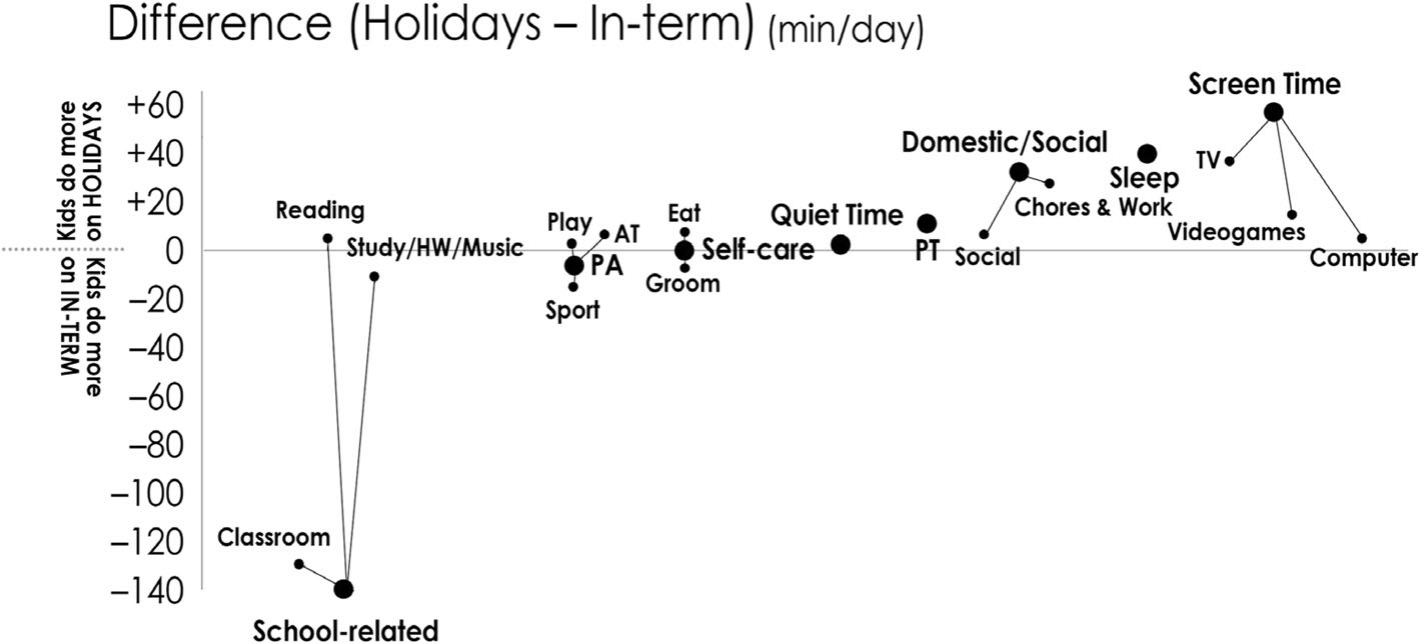
Differences in time use (min/day) between Holiday and In-term periods (Holiday minus In-term). The large dots represent superdomains, and the smaller dots dependent macrodomains. Anything above the midline indicates activities children spend more time doing in the Holiday period; anything below the mid-line is an activity children spend more time doing in the In-term period. AT = Active Transport; PA = Physical Activity; PT = Passive Transport

There was a significant interaction between time of measurement and time-use composition *ilr* coordinates (F = 119.1, *p* < 0.0001). Time use (arithmetic means) during holiday and in-term periods are shown in Table 3 and Fig. 1. A 140 min/day reduction in School-related time was compensated by 58 min/day more Screen Time (mainly TV and Videogames), 40 min/day more sleep, 35 min/day more Domestic/Social time (mainly Chores and Work), and 12 min/day more Passive Transport. In terms of energy expenditure bands, children experienced less VPA (− 10 min/day, associated with less Sport) and sedentary time (− 33 min/day). There were no differences in LPA, MPA or MVPA. Estimated TDEE was 5.4% lower during the holiday period. Additional file 1 shows the median (25%ile-75%ile) time use during in-term and holiday periods.

**Table 3:** Mean (SD) time use during in-term and holiday periods, and difference between the two periods (holiday minus in-term). All values are in min/day except TDEE (MET.min)

Superdomain	Macrodomain	In-term time	Holiday time	Difference
Domestic/Social		75 (65)	110 (131)	+ 35
	Social	20 (34)	27 (73)	+ 7
	Chores/Work	55 (56)	83 (112)	+ 28
Passive Transport		52 (32)	64 (85)	+ 12
Physical Activity		143 (76)	136 (127)	–7
	Sport	61 (55)	45 (76)	–16
	Play	38 (49)	40 (76)	+ 2
	Active Transport	44 (36)	50 (80)	+ 6
Quiet Time		77 (54)	80 (86)	+ 3
School-related		216 (80)	76 (109)	−140
	Classroom	164 (55)	31 (66)	− 133
	Study/HW/Music	31 (48)	20 (57)	−11
	Reading	22 (34)	25 (64)	+ 3
Screen Time		201 (110)	259 (174)	+ 58
	TV	135 (81)	173 (1237)	+ 38
	Computer	34 (47)	38 (82)	+ 4
	Videogames	32 (61)	48 (97)	+ 16
Self-care		99 (29)	99 (39)	0
	Eating	57 (19)	62 (29)	+ 5
	Grooming	42 (20)	37 (25)	−5
Sleep		576 (66)	616 (108)	+ 40
Energy expenditure				
TDEE (MET.min)		2405 (360)	2282 (490)	−123
TST		516 (101)	483 (162)	−33
LPA		245 (93)	245 (149)	0
MPA		64 (57)	67 (95)	+ 3
VPA		39 (43)	29 (56)	−10

*HW* homework, *LPA* light physical activity, *MPA* moderate physical activity, *MVPA* moderate-to-vigorous physical activity, *VPA* vigorous physical activity, *TDEE* total daily energy expenditure, *TST* total sedentary time

There were no significant interactions for time of measurement, time-use composition (*ilr* number) and either highest parental education level (F = 1.2, *p* = 0.26), IRSD (F = 1.4, *p* = 0.0.21) or *z*BMI (F = 1.2, *p* = 0.32).

## Discussion

### Main findings

As hypothesised, there were significant differences in activity compositions between in-term and holiday periods in Australian children. In the holiday period, to compensate for the 140 min/day reduction in School-related time, children slept for 40 min/day longer, and experienced 58 min/day more Screen Time. They also accumulated more Domestic/Social time and spent longer in Passive Transport. However, they accumulated 10 min/day less VPA. As a result, estimated TDEE fell by more than 5%. However, contrary to our hypothesis, these differences were consistent across both area- and household-level SES tertiles, with only minor, non-significant differences across weight status categories.

The findings of this study are broadly consistent with the few other studies of holiday time-use in children. Like Zinkel et al. [18], we found that TDEE was greater during in-term time than in holidays: where Zinkel et al. [18] reported a non-significant 2.4% differential, we estimated a 5.4% difference. Staiano et al. [20] reported 30 min/day more TV time during the holiday break (vs 38 min/day in the current study), and 12 min/day more computer time (vs 20 min/day for computer and videogames combined).

The lower sitting time (− 33 min/day) during the Holiday period might seem surprising, especially given the increase in screen time (+ 58 min/day). However, a large part of the school day is spent sitting, and in the Holiday period children spent 140 min/day less on school-related activities. There were also increases in time spent in Chores (+ 28 min/day), understandable since the children spent more time at home, and in passive transport (+ 12 min/day), probably reflecting holiday excursions. The increased sleep time (+ 40 min/day) was entirely due to later wake times. There were only very small differences in either eating or grooming. These obligatory activities vary within a narrow range at all ages. Quiet Time (mainly “chilling out”) was also relatively unchanged (+ 3 min/day).

There were no differences in MPA (+ 3 min/day) or LPA (0 min/day), probably due to compensatory shifts in time spent in different activity domains (for example, a 28 min/day increase in chores and a 140 min/day decrease in school-related activities). It is of interest that 78 children reported some “school-related” activity in the holidays, perhaps reflecting study, private tutoring or holiday learning camps. Because the MARCA does not identify specific contexts, activities such as “writing — sitting”, which fall under the School-related rubric, are not necessarily performed at school.

Though downstream health impacts of activity patterns, such as change in BMI and fitness, could not be determined in the current study design, the changes in activity patterns detected in our study are certainly consistent with international evidence for holiday fitness losses and weight gain. For example, VPA is strongest predictor of fitness compared with other physical activity metrics [35]. Similarly, the increase in sleeping time coupled with loss of VPA in the current study creates a net reduction in TDEE of 5.4% during holidays. If we assume that summer holiday activities reflects holiday activities across the year, then assuming a basal metabolic rate of 6334 kJ predicted by the Schofield equations for children of this age and size [36], and using the MARCA-estimated physical activity level (PAL) of 1.67 METs, a decrement of 5.4% in TDEE would equate to a deficit in EE of about 570 kJ/day, or 23,940 kJ over the six-week summer holidays. Uncompensated by dietary changes, this would result in the accumulation of about 650 g of body fat.

Contrary to expectations, the differences between in-term and holiday activity compositions were not moderated by socioeconomic status or weight status. Brazendale and colleagues [37] have recently noted that children’s activity patterns on holidays closely resembled weekend days, which they attribute to weekend days and holidays lacking daily structure, compared with school days (they call this the “Structured Day Hypothesis”). Our previous study of Australian children who participated in the National Children’s Nutrition and Physical Activity Survey (the same study from which the current subset was drawn) found that children from lower socioeconomic backgrounds experienced less sport and less vigorous physical activity that their more well-off peers, and the gap widened on weekends [38]. Based on this, we might have expected to have found socioeconomic differences in the current analysis. However, it is possible that with its more modest sample size, differences failed to reach statistical significance in the current study.

### Strengths and limitations

This is one of very few studies to capture time use during the holiday period, and to our knowledge, the first Australian study to do so. It used a valid, reliable, high-resolution use-of-time recall. In addition, the data for this study were drawn from a nationally representative sample, and the analytic subgroup used for the current study was typical of the larger sample in terms major sociodemographic characteristics (geographical distribution, parental education, adiposity, age, and sex distribution), supporting the generalisability of findings. The school term time and holiday sampling frames were well matched in terms of season (midpoint date for both was 9 June, 2007), suggesting that seasonal variation should not have biased results.

Limitations must also be acknowledged. All self-report measures are susceptible to both recall and desirability biases, minimised here by use of the 24-h day reconstruction technique. While the MARCA has been validated in a wide range of populations against several accelerometers and doubly-labeled water, there has been no specific validation for VPA, and given the intermittent pattern in which children acquire VPA, some MPA may have been misclassified as VPA. Another issue is that it is somewhat easier to recall structured days, like school days, than non-structured days (holidays and weekends) [39]. While this is unlikely to lead to systematic bias, it may reduce reliability. Furthermore, this study was conducted as a secondary analysis, and the original study was not designed specifically to examine differences between holidays and school time. It is possible that additional differences may become apparent if more holiday days had been sampled in order to better estimate “usual” holiday activities. Further to this, different patterns may emerge in a dataset specifically designed to contrast school time and various school holiday periods (e.g. summer vs winter holidays). An additional limitation is that these data were collected over a decade ago, and children’s time use may have changed appreciably since then, particularly in relation to screen time, with the development of convergent, ubiquitous, portable electronic devices. We have unpublished data comparing 11–12 year olds in 2005 to their peers in 2015 using the same recall instrument, across all day types. Interestingly, screen time decreased by about 20 min/day (entirely due to less TV), as did physical activity (mainly active transport and play). These were compensated by increases in quiet time (“chilling out”) and grooming. However, there are no data we know of showing how holiday time use specifically has changed. At most, only two days of each day type were sampled which would reduce the precision of parameter estimates, but would not be expected to bias them.

### Implications

To date, research examining the etiology of children’s weight gain and fitness losses during holidays has been scarce. In future, longitudinal research designs, simultaneously examining activity patterns, eating patterns, weight gain and fitness, are recommended. One has to be cautious in generalising from a single study, since a number of inter-country factors are likely to moderate and/or mediate the relationship between holiday weight gain and fitness loss and lifestyle. For example, the length of school holidays is highly variable (typically 6 weeks in Australia, compared with up to 14 weeks in the USA). Also, the timing of cultural festivities varies between countries (for example, in Australia, the Christmas season coincides with summer holidays, whereas it falls in winter in the northern hemisphere).

Brazendale et al. [37] reviewed 190 studies reporting activity patterns on school days and weekend days, as part of their discussion paper on the “Structured Day hypothesis”. They found that around 80% of included studies showed evidence of obesogenic behaviours being more unfavorable on weekend days compared to school days, which is likely due to the school day being segmented, pre-planned and incorporating compulsory elements (such as school-based physical activity and a large portion of the day being spent in class room activities, limiting the amount of discretionary time for unfavorable activities such as recreational screen time). This may suggest that unfavourable activity patterns and health outcomes associated with children’s holidays may be addressed by extending the school environment to holidays, through radical restructures of the school year, which have been mooted and are being trialed overseas [40], or by fostering residential or non-residential summer camps which are already widespread in Europe and North America. In France, for example, 25% of all students attend summer *colonies de vacances*. Recently there has been some discussion around establishing a culture of summer camps in Australia [41]. There is evidence that these camps, with appropriate activity programs, can be effective in promoting leanness and fitness. A recent study [42] found that disadvantaged adolescents who attended a 5-week summer camp including 1 h of PA each day reduced their body fat by 1.7%BF, and increased VO_2max_ by 3.2 ml/kg/min relative to non-attendees.

At present, most interventions directed at children’s fitness and fatness are school-based, which allows greater reach and greater equity. Although challenging, especially during the holiday diaspora, family-based interventions can be effective in increasing physical activity, with a systematic review [43] showing an overall effect size of 0.29. Another systematic review [44] found family-based interventions to be more effective than school-based interventions for reducing obesity in children under the age of 12.

## Conclusion

Activity compositions between in-term and holiday periods in Australian children differ significantly. In particular, during the holiday period, children sleep for three quarters of an hour longer, experience nearly 1 h more Screen Time, and get 10 min less VPA each day. Against a background of evidence suggesting children are losing fitness and gaining weight during the school holidays, this would suggest that interventions promoting healthful activity patterns during school holidays are warranted.

## Additional file

Additional file 1: **Table S1.** Median (25%ile-75%ile) time use during in-term and holiday periods. All values are in min/day except TDEE (MET.min). (DOCX 19 kb)

## Abbreviations

AT: Active transport
BMI: Body mass index
IRSD: Index of Relative Social Disadvantage
LPA: Light physical activity
MARCA: Multimedia Activity Recall for Children and Adults
MET: Metabolic equivalent of task
MPA: Moderate physical activity
MVPA: Moderate to vigorous physical activity
NCNPAS: National Children’s Nutrition and Physical Activity Survey
PA: Physical activity
PT: Passive transport
SEIFA: Socio-economic Indicators for Areas
TDEE: Total daily energy expenditure
VPA: Vigorous physical activity
