# Supplementary material for: Life on holidays: differences in activity composition between school and holiday periods in Australian children
Source: BMC Public Health. 2019 Jun 3;19(Suppl 2):450. doi: 10.1186/s12889-019-6765-6 (PMC6546614; doi:10.1186/s12889-019-6765-6)
Supplement: Supplementary file 1 — Table S1. Median (25%ile-75%ile) time use during in-term and holiday periods. All values are in min/day except TDEE (MET.min). (DOCX 19 kb) [file 12889_2019_6765_MOESM1_ESM.docx]

Additional file 1: Table S1. Median (25%ile-75%ile) time use during in-term and holiday periods. All values are in min/day except TDEE (MET.min).

| **Superdomain** | **Macrodomain** | **In-term time** | **Holiday time** |
| --- | --- | --- | --- |
| **Domestic/Social** |  | 58 (27-104) | 60 (15-145) |
|  | **Social** | 7 (0-26) | 0 (0-20) |
|  | **Chores/Work** | 36 (14-79) | 40 (5-110) |
| **Passive Transport** |  | 47 (26-71) | 40 (0-85) |
| **Physical Activity** |  | 132 (87-192) | 105 (30-190) |
|  | **Sport** | 50 (14-94) | 0 (0-65) |
|  | **Play** | 18 (0-56) | 0 (0-55) |
|  | **Active Transport** | 37 (18-64) | 20 (0-60) |
| **Quiet Time** |  | 65 (36-105) | 55 (10-125) |
| **School-related** |  | 212 (159-257) | 25 (0-120) |
|  | **Classroom** | 142 (107-179) | 0 (0-0) |
|  | **Study/HW/Music** | 11 (0-43) | 0 (0-0) |
|  | **Reading** | 6 (0-32) | 0 (0-10) |
| **Screen Time** |  | 183 (117-270) | 235 (130-370) |
|  | **TV** | 122 (75-185) | 165 (60-250) |
|  | **Computer** | 14 (0-51) | 0 (0-40) |
|  | **Videogames** | 7 (0-41) | 0 (0-60) |
| **Self-care** |  | 96 (79-116) | 95 (70-120) |
|  | **Eating** | 56 (44-68) | 55 (45-75) |
|  | **Grooming** | 39 (29-51) | 30 (20-50) |
| **Sleep** |  | 579 (526-621) | 625 (540-690) |
| **Energy Expenditure Bands** | | | |
| **TDEE (MET.min)** |  | 2330 (2147-2613) | 2177 (1915-2559) |
| **TST** |  | 509 (447-584) | 485 (370-580) |
| **LPA** |  | 236 (180-301) | 223 (130-230) |
| **MPA** |  | 52 (23-87) | 30 (0-90) |
| **VPA** |  | 25 (4-59) | 0 (0-30) |

HW = homework; LPA = light physical activity; MPA = moderate physical activity; MVPA = moderate-to-vigorous physical activity; VPA = vigorous physical activity; TDEE = total daily energy expenditure; TST = total sedentary time
